# 2-Dimethylamino-1-(2-ethoxy-2-oxo­ethyl)-3-methyl-3,4,5,6-tetrahydro­pyrimidin-1-ium tetraphenylborate

**DOI:** 10.1107/S1600536812023951

**Published:** 2012-06-02

**Authors:** Ioannis Tiritiris, Willi Kantlehner

**Affiliations:** a Institut für Organische Chemie, Universität Stuttgart, Pfaffenwaldring 55, 70569 Stuttgart, Germany; b Fakultät Chemie/Organische Chemie, Hochschule Aalen, Beethovenstrasse 1, D-73430 Aalen, Germany

## Abstract

Isolated guanidinium ions and tetra­phenyl­borate ions are present in the crystal structure of the title compound, C_11_H_22_N_3_O_2_^+^·C_24_H_20_B^−^. In the guanidinium ion, the dihedral angle between the N/C/N and C/C/C planes being 49.9 (1)°. The six-membered ring exhibits a half-chair conformation. The C—N bond lengths in the cation range between 1.3335 (16) and 1.3552 (16) Å, indicating charge delocalization on the CN_3_ plane. In the crystal, the cations are connected by C—H⋯O hydrogen bonds, generating a chain along the *c* axis.

## Related literature

For the synthesis and nematocidal activity of aryl­vinyl­tetra­hydro­pyrimidines, see: Kraouti *et al.* (1993 ▶). For the synthesis and nematocidal activity of pyran­tel analogs, see: Kraouti *et al.* (1995 ▶). For the synthesis of 1-methyl-2-dimethyl­amino-1,4,5,6-tetra­hydro­pyrimidine and derived cyclic guanidinium salts, see: Tiritiris & Kantlehner (2012 ▶).
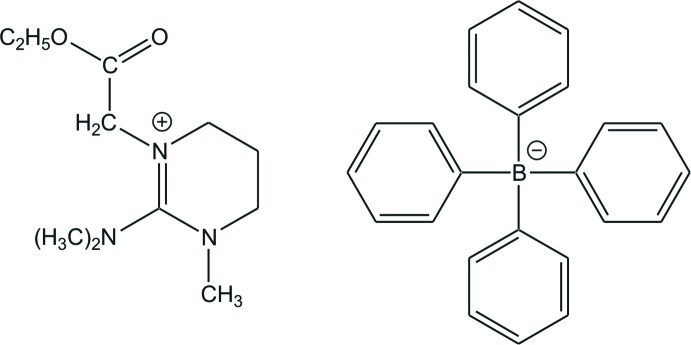


## Experimental

### 

#### Crystal data

C_11_H_22_N_3_O_2_^+^·C_24_H_20_B^−^
*M* *_r_* = 547.53
Monoclinic, 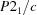

*a* = 14.3582 (5) Å
*b* = 10.3377 (3) Å
*c* = 20.6302 (9) Å
β = 105.615 (1)°
*V* = 2949.14 (19) Å^3^
*Z* = 4
Mo *K*α radiation
μ = 0.08 mm^−1^
*T* = 100 K
0.23 × 0.16 × 0.13 mm

#### Data collection

Bruker–Nonius Kappa CCD diffractometer
13034 measured reflections
6747 independent reflections
5041 reflections with *I* > 2σ(*I*)
*R*_int_ = 0.035

#### Refinement

*R*[*F* ^2^ > 2σ(*F* ^2^)] = 0.040
*wR*(*F* ^2^) = 0.097
*S* = 1.02
6747 reflections
374 parameters
H-atom parameters constrained
Δρ_max_ = 0.30 e Å^−3^
Δρ_min_ = −0.21 e Å^−3^

### 

Data collection: *COLLECT* (Hooft, 2004 ▶); cell refinement: *HKL* *SCALEPACK* (Otwinowski & Minor, 1997 ▶); data reduction: *HKL* *SCALEPACK*; program(s) used to solve structure: *SHELXS97* (Sheldrick, 2008 ▶); program(s) used to refine structure: *SHELXL97* (Sheldrick, 2008 ▶); molecular graphics: *DIAMOND* (Brandenburg & Putz, 2005 ▶); software used to prepare material for publication: *SHELXL97*.

## Supplementary Material

Crystal structure: contains datablock(s) I, global. DOI: 10.1107/S1600536812023951/kp2422sup1.cif

Structure factors: contains datablock(s) I. DOI: 10.1107/S1600536812023951/kp2422Isup2.hkl

Additional supplementary materials:  crystallographic information; 3D view; checkCIF report

## Figures and Tables

**Table 1:** Hydrogen-bond geometry (Å, °)

*D*—H⋯*A*	*D*—H	H⋯*A*	*D*⋯*A*	*D*—H⋯*A*
C10—H10*B*⋯O1^i^	0.99	2.44	3.397 (2)	163

Symmetry code: (i) 
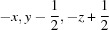
.

**Fig. 1.**
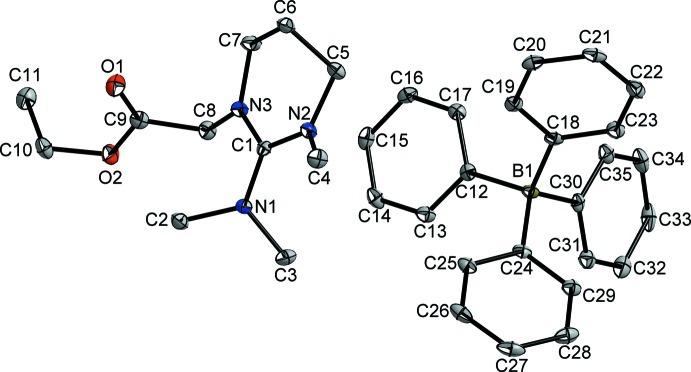
The structure of the title compound with atom labels and 50% probability displacement ellipsoids. All H atoms have been omitted for clarity.

**Fig. 2.**
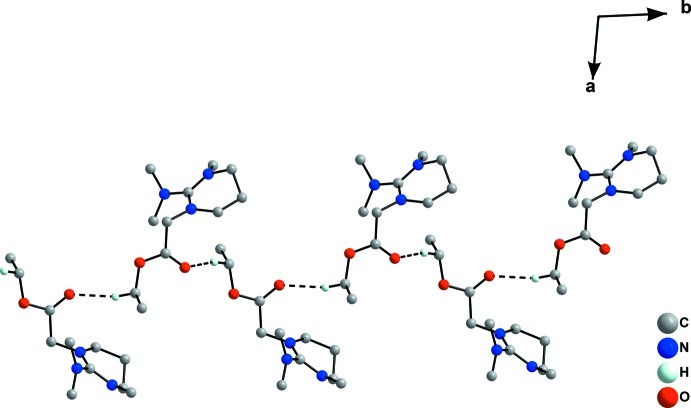
C—H···O hydrogen bonds between the guanidinium ions in ab-view. The hydrogen bonds are indicated by dashed lines.

## supplementary crystallographic information

### Comment

Tetrahydropyrimidine derivatives very often show pharmacologic activity. Prominent members are the cyclic amidines oxantel (Kraouti *et al.*, 1993) and pyrantel (Kraouti *et al.*, 1995), which are showing an anthelmintic effect against intestinal nematode infestations in humans and animals. 1-Methyl-2-dimethylamino-1,4,5,6-tetrahydropyrimidine (Tiritiris & Kantlehner, 2012), a cyclic guanidine derivative synthesized by us recently, could be used as a new candidate for preparing potentially pharmacologically active compounds in this field. By alkylation of the free nitrogen of the guanidine base, various cyclic guanidinium salts have been obtained and characterised (Tiritiris & Kantlehner, 2012). One of them, is the here presented title compound (Fig. 1). In the crystal structure of the salt,

isolated cations and anions are present. No specific interactions between the guanidinium ions and the tetraphenylborate ions have been observed. Prominent bond parameters in the guanidinium ion are: C1–N1 = 1.355 (2) Å, C1–N2 = 1.334 (2) Å and C1–N3 = 1.343 (2) Å. The N–C1–N angles are: 120.0 (1)° (N1–C1–N2), 120.3 (1)° (N2–C1–N3) and 119.8 (1)° (N1–C1–N3), which indicates a nearly ideal trigonal-planar surrounding of the carbon centre by the nitrogen atoms. The positive charge is completely delocalized on the CN_3_ plane. Bond lengths between carbon and oxygen atoms in the ethoxycarbonylmethyl group are: C9–O1 = 1.206 (2) Å, C9–O2 = 1.333 (2) Å and C10–O2 = 1.467 (2) Å. The six membered ring is non planar (Fig. 1). The carbon atom C6 is not in the ring plane, the angle between the planes N3/C1/N2 and C5/C6/C7 is 49.9 (1)°. Finally, weak C–H···O hydrogen bonds between methylene hydrogen atoms and carbonyl oxygen atoms of neighbouring guanidinium ions have been determined [*d*(H···O) = 2.44 Å] (Tab. 1). The cations are connected by C–H···O hydrogen bonds, generating a chain (Fig. 2). The anions are packed inbetween these chains using van der Waals interactions, only.

### Experimental

The title compound was obtained by reaction of 1-methyl-2-dimethylamino-1,4,5,6-tetrahydropyrimidine with bromoacetic acid ethyl ester in acetonitrile at room temperature. After evaporation of the solvent the crude 2-dimethylamino-3-ethoxycarbonylmethyl-1-methyl-1,4,5,6- tetrahydropyrimidinium-bromide (I) was washed with diethylether and dried *in vacuo*. 1.05 g (3.4 mmol) of (I) was dissolved in 20 mL acetonitrile and 1.16 g (3.4 mmol) of sodium tetraphenylborate in 10 mL acetonitrile was added. After stirring for one h at room temperature, the precipitated sodium bromide was filtered off. The title compound crystallised from a saturated acetonitrile solution after several days at 273 K, forming colourless single crystals. Yield: 1.43 g (78.8%).

### Refinement

The hydrogen atoms of the methyl groups were allowed to rotate with a fixed angle around the C–N bond to best fit the experimental electron density, with *U*(H) set to 1.5 *U*_eq_(C) and d(C—H) = 0.98 Å. The remaining H atoms were placed in calculated positions with d(C—H) = 0.99 Å (H atoms in CH_2_ groups) and (C—H) = 0.95 Å (H atoms in aromatic rings). They were included in the refinement in the riding model approximation, with *U*(H) set to 1.2 *U*_eq_(C).

### Crystal data

**Table:**

C_11_H_22_N_3_O_2_^+^·C_24_H_20_B^−^	*F*(000) = 1176
*M**_r_* = 547.53	*D*_x_ = 1.233 Mg m^−^^3^
Monoclinic, *P*2_1_/*c*	Melting point: 458 K
Hall symbol: -P 2ybc	Mo *K*α radiation, λ = 0.71073 Å
*a* = 14.3582 (5) Å	Cell parameters from 7081 reflections
*b* = 10.3377 (3) Å	θ = 0.4–27.5°
*c* = 20.6302 (9) Å	µ = 0.08 mm^−^^1^
β = 105.615 (1)°	*T* = 100 K
*V* = 2949.14 (19) Å^3^	Polyhedral, colourless
*Z* = 4	0.23 × 0.16 × 0.13 mm

### Data collection

**Table:**

Bruker–Nonius Kappa CCD diffractometer	5041 reflections with *I* > 2σ(*I*)
Radiation source: sealed tube	*R*_int_ = 0.035
Graphite monochromator	θ_max_ = 27.5°, θ_min_ = 1.5°
φ scans, and ω scans	*h* = −18→18
13034 measured reflections	*k* = −13→13
6747 independent reflections	*l* = −26→26

### Refinement

**Table:**

Refinement on *F*^2^	Primary atom site location: structure-invariant direct methods
Least-squares matrix: full	Secondary atom site location: difference Fourier map
*R*[*F*^2^ > 2σ(*F*^2^)] = 0.040	Hydrogen site location: difference Fourier map
*wR*(*F*^2^) = 0.097	H-atom parameters constrained
*S* = 1.02	*w* = 1/[σ^2^(*F*_o_^2^) + (0.0412*P*)^2^ + 0.9044*P*] where *P* = (*F*_o_^2^ + 2*F*_c_^2^)/3
6747 reflections	(Δ/σ)_max_ < 0.001
374 parameters	Δρ_max_ = 0.30 e Å^−^^3^
0 restraints	Δρ_min_ = −0.21 e Å^−^^3^

### Special details

**Table:**

Geometry. All e.s.d.'s (except the e.s.d. in the dihedral angle between two l.s. planes) are estimated using the full covariance matrix. The cell e.s.d.'s are taken into account individually in the estimation of e.s.d.'s in distances, angles and torsion angles; correlations between e.s.d.'s in cell parameters are only used when they are defined by crystal symmetry. An approximate (isotropic) treatment of cell e.s.d.'s is used for estimating e.s.d.'s involving l.s. planes.
Refinement. Refinement of *F*^2^ against ALL reflections. The weighted *R*-factor *wR* and goodness of fit *S* are based on *F*^2^, conventional *R*-factors *R* are based on *F*, with *F* set to zero for negative *F*^2^. The threshold expression of *F*^2^ > σ(*F*^2^) is used only for calculating *R*-factors(gt) *etc*. and is not relevant to the choice of reflections for refinement. *R*-factors based on *F*^2^ are statistically about twice as large as those based on *F*, and *R*- factors based on ALL data will be even larger.

### Fractional atomic coordinates and isotropic or equivalent isotropic displacement parameters (Å^2^)

**Table:**

	*x*	*y*	*z*	*U*_iso_*/*U*_eq_	
C1	0.28958 (8)	0.25769 (11)	0.34347 (6)	0.0115 (3)	
N1	0.27143 (7)	0.17051 (10)	0.38759 (5)	0.0139 (2)	
C2	0.17367 (9)	0.12719 (13)	0.38452 (7)	0.0175 (3)	
H2A	0.1273	0.1724	0.3480	0.026*	
H2B	0.1593	0.1462	0.4273	0.026*	
H2C	0.1687	0.0338	0.3762	0.026*	
C3	0.34770 (9)	0.12482 (13)	0.44516 (7)	0.0173 (3)	
H3A	0.4109	0.1504	0.4397	0.026*	
H3B	0.3446	0.0303	0.4479	0.026*	
H3C	0.3388	0.1631	0.4866	0.026*	
N2	0.34721 (7)	0.35845 (10)	0.36596 (5)	0.0130 (2)	
C4	0.35502 (10)	0.41502 (13)	0.43243 (6)	0.0175 (3)	
H4A	0.3116	0.3692	0.4540	0.026*	
H4B	0.3369	0.5066	0.4273	0.026*	
H4C	0.4218	0.4071	0.4604	0.026*	
C5	0.37840 (9)	0.44036 (12)	0.31723 (6)	0.0151 (3)	
H5A	0.4279	0.3948	0.3005	0.018*	
H5B	0.4068	0.5219	0.3390	0.018*	
C6	0.29063 (9)	0.46922 (12)	0.25951 (7)	0.0163 (3)	
H6A	0.2405	0.5133	0.2764	0.020*	
H6B	0.3087	0.5266	0.2264	0.020*	
C7	0.25227 (10)	0.34237 (13)	0.22685 (6)	0.0177 (3)	
H7A	0.1860	0.3555	0.1975	0.021*	
H7B	0.2935	0.3128	0.1983	0.021*	
N3	0.25016 (7)	0.24143 (10)	0.27716 (5)	0.0130 (2)	
C8	0.21616 (9)	0.11507 (12)	0.24902 (6)	0.0146 (3)	
H8A	0.2400	0.0478	0.2837	0.017*	
H8B	0.2435	0.0966	0.2108	0.017*	
C9	0.10628 (9)	0.10783 (12)	0.22489 (6)	0.0147 (3)	
O1	0.05354 (6)	0.20070 (9)	0.21392 (5)	0.0202 (2)	
O2	0.07769 (6)	−0.01524 (9)	0.21815 (5)	0.0186 (2)	
C10	−0.02683 (9)	−0.03720 (13)	0.19344 (7)	0.0199 (3)	
H10A	−0.0618	0.0332	0.2094	0.024*	
H10B	−0.0437	−0.1199	0.2117	0.024*	
C11	−0.05773 (10)	−0.04170 (14)	0.11791 (7)	0.0229 (3)	
H11A	−0.0420	0.0407	0.0999	0.034*	
H11B	−0.1276	−0.0566	0.1024	0.034*	
H11C	−0.0237	−0.1121	0.1021	0.034*	
B1	0.72345 (10)	0.26120 (14)	0.43053 (7)	0.0121 (3)	
C12	0.62846 (8)	0.20787 (11)	0.37300 (6)	0.0118 (2)	
C13	0.57673 (9)	0.09718 (12)	0.38220 (6)	0.0158 (3)	
H13A	0.5935	0.0560	0.4249	0.019*	
C14	0.50187 (9)	0.04537 (13)	0.33128 (7)	0.0181 (3)	
H14A	0.4681	−0.0289	0.3400	0.022*	
C15	0.47629 (9)	0.10144 (13)	0.26797 (7)	0.0162 (3)	
H15A	0.4251	0.0666	0.2331	0.019*	
C16	0.52690 (9)	0.20960 (12)	0.25643 (6)	0.0151 (3)	
H16A	0.5109	0.2487	0.2132	0.018*	
C17	0.60097 (9)	0.26084 (12)	0.30805 (6)	0.0131 (3)	
H17A	0.6344	0.3350	0.2989	0.016*	
C18	0.73770 (9)	0.41831 (12)	0.42497 (6)	0.0126 (3)	
C19	0.65918 (9)	0.50381 (12)	0.40846 (6)	0.0142 (3)	
H19A	0.5956	0.4691	0.3960	0.017*	
C20	0.67052 (10)	0.63761 (12)	0.40953 (6)	0.0165 (3)	
H20A	0.6152	0.6920	0.3976	0.020*	
C21	0.76223 (10)	0.69196 (12)	0.42790 (6)	0.0175 (3)	
H21A	0.7703	0.7832	0.4288	0.021*	
C22	0.84182 (10)	0.61043 (13)	0.44488 (7)	0.0184 (3)	
H22A	0.9052	0.6459	0.4577	0.022*	
C23	0.82928 (9)	0.47688 (12)	0.44329 (6)	0.0158 (3)	
H23A	0.8850	0.4231	0.4551	0.019*	
C24	0.71533 (9)	0.24075 (11)	0.50776 (6)	0.0123 (3)	
C25	0.62668 (9)	0.24171 (12)	0.52421 (7)	0.0152 (3)	
H25A	0.5687	0.2474	0.4888	0.018*	
C26	0.62008 (10)	0.23463 (12)	0.59031 (7)	0.0193 (3)	
H26A	0.5584	0.2343	0.5990	0.023*	
C27	0.70307 (11)	0.22805 (13)	0.64337 (7)	0.0215 (3)	
H27A	0.6989	0.2225	0.6885	0.026*	
C28	0.79239 (10)	0.22970 (13)	0.62940 (7)	0.0201 (3)	
H28A	0.8500	0.2269	0.6652	0.024*	
C29	0.79771 (10)	0.23546 (12)	0.56298 (6)	0.0158 (3)	
H29A	0.8597	0.2358	0.5547	0.019*	
C30	0.81220 (8)	0.17917 (12)	0.41432 (6)	0.0126 (3)	
C31	0.84580 (9)	0.06116 (12)	0.44527 (7)	0.0161 (3)	
H31A	0.8215	0.0319	0.4812	0.019*	
C32	0.91335 (9)	−0.01510 (13)	0.42549 (7)	0.0198 (3)	
H32A	0.9350	−0.0938	0.4484	0.024*	
C33	0.94894 (9)	0.02374 (13)	0.37262 (7)	0.0217 (3)	
H33A	0.9941	−0.0285	0.3583	0.026*	
C34	0.91763 (9)	0.14018 (14)	0.34078 (7)	0.0201 (3)	
H34A	0.9418	0.1683	0.3046	0.024*	
C35	0.85121 (9)	0.21571 (13)	0.36156 (6)	0.0158 (3)	
H35A	0.8313	0.2954	0.3392	0.019*	

### Atomic displacement parameters (Å^2^)

**Table:**

	*U*^11^	*U*^22^	*U*^33^	*U*^12^	*U*^13^	*U*^23^
C1	0.0109 (6)	0.0104 (6)	0.0133 (6)	0.0025 (5)	0.0037 (5)	0.0003 (5)
N1	0.0139 (5)	0.0132 (5)	0.0138 (5)	−0.0011 (4)	0.0025 (4)	0.0033 (4)
C2	0.0178 (6)	0.0163 (6)	0.0195 (7)	−0.0016 (5)	0.0071 (5)	0.0022 (5)
C3	0.0208 (7)	0.0149 (6)	0.0146 (6)	0.0026 (5)	0.0021 (5)	0.0031 (5)
N2	0.0155 (5)	0.0122 (5)	0.0115 (5)	−0.0021 (4)	0.0040 (4)	−0.0007 (4)
C4	0.0224 (7)	0.0155 (6)	0.0139 (6)	−0.0024 (5)	0.0039 (5)	−0.0038 (5)
C5	0.0168 (6)	0.0133 (6)	0.0164 (6)	−0.0025 (5)	0.0066 (5)	0.0002 (5)
C6	0.0183 (6)	0.0143 (6)	0.0172 (6)	−0.0007 (5)	0.0065 (5)	0.0035 (5)
C7	0.0229 (7)	0.0176 (7)	0.0116 (6)	−0.0019 (5)	0.0030 (5)	0.0027 (5)
N3	0.0160 (5)	0.0109 (5)	0.0115 (5)	−0.0011 (4)	0.0027 (4)	−0.0005 (4)
C8	0.0163 (6)	0.0119 (6)	0.0150 (6)	−0.0011 (5)	0.0034 (5)	−0.0032 (5)
C9	0.0189 (6)	0.0131 (6)	0.0119 (6)	−0.0019 (5)	0.0041 (5)	−0.0031 (5)
O1	0.0177 (5)	0.0153 (5)	0.0266 (5)	0.0016 (4)	0.0041 (4)	−0.0034 (4)
O2	0.0182 (5)	0.0145 (5)	0.0209 (5)	−0.0037 (4)	0.0015 (4)	−0.0023 (4)
C10	0.0180 (7)	0.0197 (7)	0.0228 (7)	−0.0063 (5)	0.0070 (5)	−0.0031 (6)
C11	0.0200 (7)	0.0246 (7)	0.0226 (7)	−0.0001 (6)	0.0032 (6)	−0.0010 (6)
B1	0.0146 (7)	0.0124 (7)	0.0094 (7)	−0.0008 (5)	0.0033 (5)	0.0003 (5)
C12	0.0124 (6)	0.0109 (6)	0.0130 (6)	0.0014 (5)	0.0050 (5)	−0.0020 (5)
C13	0.0202 (7)	0.0147 (6)	0.0126 (6)	−0.0012 (5)	0.0044 (5)	0.0008 (5)
C14	0.0197 (7)	0.0151 (6)	0.0210 (7)	−0.0062 (5)	0.0083 (5)	−0.0046 (5)
C15	0.0127 (6)	0.0186 (7)	0.0165 (6)	0.0001 (5)	0.0026 (5)	−0.0074 (5)
C16	0.0178 (6)	0.0158 (6)	0.0115 (6)	0.0037 (5)	0.0036 (5)	−0.0006 (5)
C17	0.0151 (6)	0.0107 (6)	0.0145 (6)	0.0005 (5)	0.0055 (5)	−0.0011 (5)
C18	0.0180 (6)	0.0132 (6)	0.0069 (6)	−0.0009 (5)	0.0040 (5)	−0.0002 (5)
C19	0.0165 (6)	0.0156 (6)	0.0103 (6)	−0.0015 (5)	0.0033 (5)	−0.0014 (5)
C20	0.0227 (7)	0.0149 (6)	0.0114 (6)	0.0041 (5)	0.0036 (5)	0.0002 (5)
C21	0.0308 (7)	0.0115 (6)	0.0107 (6)	−0.0032 (5)	0.0066 (5)	−0.0003 (5)
C22	0.0198 (7)	0.0185 (7)	0.0174 (7)	−0.0065 (5)	0.0059 (5)	−0.0013 (5)
C23	0.0172 (6)	0.0145 (6)	0.0154 (6)	−0.0004 (5)	0.0038 (5)	−0.0001 (5)
C24	0.0182 (6)	0.0062 (5)	0.0129 (6)	−0.0008 (5)	0.0049 (5)	−0.0005 (5)
C25	0.0192 (6)	0.0103 (6)	0.0172 (6)	−0.0018 (5)	0.0068 (5)	−0.0009 (5)
C26	0.0273 (7)	0.0120 (6)	0.0237 (7)	−0.0019 (5)	0.0157 (6)	−0.0006 (5)
C27	0.0407 (8)	0.0136 (6)	0.0133 (6)	0.0003 (6)	0.0127 (6)	0.0000 (5)
C28	0.0299 (8)	0.0149 (6)	0.0129 (6)	0.0007 (6)	0.0013 (5)	−0.0009 (5)
C29	0.0187 (6)	0.0133 (6)	0.0152 (6)	−0.0012 (5)	0.0044 (5)	−0.0004 (5)
C30	0.0116 (6)	0.0133 (6)	0.0122 (6)	−0.0039 (5)	0.0017 (5)	−0.0042 (5)
C31	0.0164 (6)	0.0149 (6)	0.0167 (7)	−0.0018 (5)	0.0038 (5)	−0.0013 (5)
C32	0.0162 (6)	0.0143 (6)	0.0265 (7)	0.0004 (5)	0.0017 (5)	−0.0036 (6)
C33	0.0123 (6)	0.0207 (7)	0.0325 (8)	−0.0017 (5)	0.0066 (6)	−0.0124 (6)
C34	0.0169 (7)	0.0242 (7)	0.0219 (7)	−0.0063 (6)	0.0098 (5)	−0.0071 (6)
C35	0.0159 (6)	0.0154 (6)	0.0162 (6)	−0.0036 (5)	0.0044 (5)	−0.0031 (5)

### Geometric parameters (Å, º)

**Table:**

C1—N2	1.3335 (16)	C12—C13	1.4047 (17)
C1—N3	1.3427 (16)	C13—C14	1.3921 (18)
C1—N1	1.3552 (16)	C13—H13A	0.9500
N1—C2	1.4584 (16)	C14—C15	1.3851 (19)
N1—C3	1.4604 (16)	C14—H14A	0.9500
C2—H2A	0.9800	C15—C16	1.3886 (18)
C2—H2B	0.9800	C15—H15A	0.9500
C2—H2C	0.9800	C16—C17	1.3914 (17)
C3—H3A	0.9800	C16—H16A	0.9500
C3—H3B	0.9800	C17—H17A	0.9500
C3—H3C	0.9800	C18—C19	1.4005 (18)
N2—C4	1.4672 (16)	C18—C23	1.4040 (18)
N2—C5	1.4732 (16)	C19—C20	1.3923 (18)
C4—H4A	0.9800	C19—H19A	0.9500
C4—H4B	0.9800	C20—C21	1.3872 (19)
C4—H4C	0.9800	C20—H20A	0.9500
C5—C6	1.5123 (18)	C21—C22	1.3869 (19)
C5—H5A	0.9900	C21—H21A	0.9500
C5—H5B	0.9900	C22—C23	1.3916 (18)
C6—C7	1.5091 (18)	C22—H22A	0.9500
C6—H6A	0.9900	C23—H23A	0.9500
C6—H6B	0.9900	C24—C25	1.4028 (18)
C7—N3	1.4779 (16)	C24—C29	1.4052 (18)
C7—H7A	0.9900	C25—C26	1.3940 (19)
C7—H7B	0.9900	C25—H25A	0.9500
N3—C8	1.4592 (15)	C26—C27	1.386 (2)
C8—C9	1.5231 (18)	C26—H26A	0.9500
C8—H8A	0.9900	C27—C28	1.388 (2)
C8—H8B	0.9900	C27—H27A	0.9500
C9—O1	1.2058 (16)	C28—C29	1.3939 (19)
C9—O2	1.3326 (15)	C28—H28A	0.9500
O2—C10	1.4669 (16)	C29—H29A	0.9500
C10—C11	1.5017 (19)	C30—C31	1.4010 (18)
C10—H10A	0.9900	C30—C35	1.4033 (18)
C10—H10B	0.9900	C31—C32	1.3934 (19)
C11—H11A	0.9800	C31—H31A	0.9500
C11—H11B	0.9800	C32—C33	1.383 (2)
C11—H11C	0.9800	C32—H32A	0.9500
B1—C30	1.6383 (18)	C33—C34	1.387 (2)
B1—C24	1.6422 (18)	C33—H33A	0.9500
B1—C12	1.6436 (18)	C34—C35	1.3868 (19)
B1—C18	1.6449 (18)	C34—H34A	0.9500
C12—C17	1.4023 (17)	C35—H35A	0.9500
			
N2—C1—N3	120.28 (11)	C24—B1—C18	103.73 (10)
N2—C1—N1	119.95 (11)	C12—B1—C18	112.05 (10)
N3—C1—N1	119.77 (11)	C17—C12—C13	115.10 (11)
C1—N1—C2	122.14 (10)	C17—C12—B1	121.63 (11)
C1—N1—C3	121.58 (10)	C13—C12—B1	122.81 (11)
C2—N1—C3	115.99 (10)	C14—C13—C12	122.71 (12)
N1—C2—H2A	109.5	C14—C13—H13A	118.6
N1—C2—H2B	109.5	C12—C13—H13A	118.6
H2A—C2—H2B	109.5	C15—C14—C13	120.37 (12)
N1—C2—H2C	109.5	C15—C14—H14A	119.8
H2A—C2—H2C	109.5	C13—C14—H14A	119.8
H2B—C2—H2C	109.5	C14—C15—C16	118.71 (12)
N1—C3—H3A	109.5	C14—C15—H15A	120.6
N1—C3—H3B	109.5	C16—C15—H15A	120.6
H3A—C3—H3B	109.5	C15—C16—C17	120.19 (12)
N1—C3—H3C	109.5	C15—C16—H16A	119.9
H3A—C3—H3C	109.5	C17—C16—H16A	119.9
H3B—C3—H3C	109.5	C16—C17—C12	122.90 (12)
C1—N2—C4	121.38 (11)	C16—C17—H17A	118.5
C1—N2—C5	119.05 (10)	C12—C17—H17A	118.5
C4—N2—C5	116.61 (10)	C19—C18—C23	115.32 (11)
N2—C4—H4A	109.5	C19—C18—B1	122.21 (11)
N2—C4—H4B	109.5	C23—C18—B1	122.14 (11)
H4A—C4—H4B	109.5	C20—C19—C18	122.69 (12)
N2—C4—H4C	109.5	C20—C19—H19A	118.7
H4A—C4—H4C	109.5	C18—C19—H19A	118.7
H4B—C4—H4C	109.5	C21—C20—C19	120.34 (12)
N2—C5—C6	107.98 (10)	C21—C20—H20A	119.8
N2—C5—H5A	110.1	C19—C20—H20A	119.8
C6—C5—H5A	110.1	C22—C21—C20	118.68 (12)
N2—C5—H5B	110.1	C22—C21—H21A	120.7
C6—C5—H5B	110.1	C20—C21—H21A	120.7
H5A—C5—H5B	108.4	C21—C22—C23	120.29 (12)
C7—C6—C5	107.87 (10)	C21—C22—H22A	119.9
C7—C6—H6A	110.1	C23—C22—H22A	119.9
C5—C6—H6A	110.1	C22—C23—C18	122.68 (12)
C7—C6—H6B	110.1	C22—C23—H23A	118.7
C5—C6—H6B	110.1	C18—C23—H23A	118.7
H6A—C6—H6B	108.4	C25—C24—C29	115.13 (12)
N3—C7—C6	111.95 (10)	C25—C24—B1	122.68 (11)
N3—C7—H7A	109.2	C29—C24—B1	121.83 (11)
C6—C7—H7A	109.2	C26—C25—C24	122.77 (12)
N3—C7—H7B	109.2	C26—C25—H25A	118.6
C6—C7—H7B	109.2	C24—C25—H25A	118.6
H7A—C7—H7B	107.9	C27—C26—C25	120.33 (13)
C1—N3—C8	121.44 (10)	C27—C26—H26A	119.8
C1—N3—C7	122.99 (10)	C25—C26—H26A	119.8
C8—N3—C7	114.86 (10)	C26—C27—C28	118.76 (12)
N3—C8—C9	112.35 (10)	C26—C27—H27A	120.6
N3—C8—H8A	109.1	C28—C27—H27A	120.6
C9—C8—H8A	109.1	C27—C28—C29	120.18 (13)
N3—C8—H8B	109.1	C27—C28—H28A	119.9
C9—C8—H8B	109.1	C29—C28—H28A	119.9
H8A—C8—H8B	107.9	C28—C29—C24	122.80 (13)
O1—C9—O2	125.47 (12)	C28—C29—H29A	118.6
O1—C9—C8	124.41 (11)	C24—C29—H29A	118.6
O2—C9—C8	110.12 (10)	C31—C30—C35	115.31 (12)
C9—O2—C10	116.21 (10)	C31—C30—B1	123.30 (11)
O2—C10—C11	110.74 (11)	C35—C30—B1	120.91 (11)
O2—C10—H10A	109.5	C32—C31—C30	122.73 (13)
C11—C10—H10A	109.5	C32—C31—H31A	118.6
O2—C10—H10B	109.5	C30—C31—H31A	118.6
C11—C10—H10B	109.5	C33—C32—C31	120.05 (13)
H10A—C10—H10B	108.1	C33—C32—H32A	120.0
C10—C11—H11A	109.5	C31—C32—H32A	120.0
C10—C11—H11B	109.5	C32—C33—C34	118.95 (13)
H11A—C11—H11B	109.5	C32—C33—H33A	120.5
C10—C11—H11C	109.5	C34—C33—H33A	120.5
H11A—C11—H11C	109.5	C35—C34—C33	120.29 (13)
H11B—C11—H11C	109.5	C35—C34—H34A	119.9
C30—B1—C24	113.19 (10)	C33—C34—H34A	119.9
C30—B1—C12	102.64 (10)	C34—C35—C30	122.65 (12)
C24—B1—C12	113.30 (10)	C34—C35—H35A	118.7
C30—B1—C18	112.26 (10)	C30—C35—H35A	118.7
			
N2—C1—N1—C2	131.82 (12)	C12—B1—C18—C19	37.60 (16)
N3—C1—N1—C2	−48.86 (17)	C30—B1—C18—C23	−34.35 (16)
N2—C1—N1—C3	−41.71 (17)	C24—B1—C18—C23	88.19 (13)
N3—C1—N1—C3	137.61 (12)	C12—B1—C18—C23	−149.24 (11)
N3—C1—N2—C4	151.00 (12)	C23—C18—C19—C20	0.44 (18)
N1—C1—N2—C4	−29.68 (17)	B1—C18—C19—C20	174.04 (12)
N3—C1—N2—C5	−8.90 (17)	C18—C19—C20—C21	−0.5 (2)
N1—C1—N2—C5	170.42 (11)	C19—C20—C21—C22	0.17 (19)
C1—N2—C5—C6	46.02 (15)	C20—C21—C22—C23	0.09 (19)
C4—N2—C5—C6	−114.81 (12)	C21—C22—C23—C18	−0.1 (2)
N2—C5—C6—C7	−61.58 (13)	C19—C18—C23—C22	−0.17 (18)
C5—C6—C7—N3	43.84 (15)	B1—C18—C23—C22	−173.77 (12)
N2—C1—N3—C8	158.29 (11)	C30—B1—C24—C25	−146.70 (11)
N1—C1—N3—C8	−21.04 (17)	C12—B1—C24—C25	−30.36 (16)
N2—C1—N3—C7	−11.55 (18)	C18—B1—C24—C25	91.38 (13)
N1—C1—N3—C7	169.12 (11)	C30—B1—C24—C29	40.54 (16)
C6—C7—N3—C1	−8.01 (17)	C12—B1—C24—C29	156.87 (11)
C6—C7—N3—C8	−178.46 (11)	C18—B1—C24—C29	−81.39 (13)
C1—N3—C8—C9	108.96 (13)	C29—C24—C25—C26	−1.56 (18)
C7—N3—C8—C9	−80.43 (13)	B1—C24—C25—C26	−174.78 (11)
N3—C8—C9—O1	17.68 (18)	C24—C25—C26—C27	0.92 (19)
N3—C8—C9—O2	−162.04 (10)	C25—C26—C27—C28	0.51 (19)
O1—C9—O2—C10	1.81 (19)	C26—C27—C28—C29	−1.2 (2)
C8—C9—O2—C10	−178.48 (10)	C27—C28—C29—C24	0.5 (2)
C9—O2—C10—C11	88.51 (14)	C25—C24—C29—C28	0.87 (18)
C30—B1—C12—C17	−84.93 (13)	B1—C24—C29—C28	174.15 (11)
C24—B1—C12—C17	152.66 (11)	C24—B1—C30—C31	30.10 (16)
C18—B1—C12—C17	35.71 (16)	C12—B1—C30—C31	−92.38 (13)
C30—B1—C12—C13	86.92 (14)	C18—B1—C30—C31	147.12 (11)
C24—B1—C12—C13	−35.48 (16)	C24—B1—C30—C35	−158.21 (11)
C18—B1—C12—C13	−152.44 (11)	C12—B1—C30—C35	79.31 (13)
C17—C12—C13—C14	−1.62 (18)	C18—B1—C30—C35	−41.18 (15)
B1—C12—C13—C14	−173.97 (12)	C35—C30—C31—C32	0.13 (18)
C12—C13—C14—C15	1.1 (2)	B1—C30—C31—C32	172.25 (12)
C13—C14—C15—C16	0.21 (19)	C30—C31—C32—C33	−1.0 (2)
C14—C15—C16—C17	−0.78 (19)	C31—C32—C33—C34	1.1 (2)
C15—C16—C17—C12	0.13 (19)	C32—C33—C34—C35	−0.39 (19)
C13—C12—C17—C16	1.04 (18)	C33—C34—C35—C30	−0.5 (2)
B1—C12—C17—C16	173.48 (11)	C31—C30—C35—C34	0.65 (18)
C30—B1—C18—C19	152.49 (11)	B1—C30—C35—C34	−171.68 (12)
C24—B1—C18—C19	−84.97 (13)		

### Hydrogen-bond geometry (Å, º)

**Table:**

*D*—H···*A*	*D*—H	H···*A*	*D*···*A*	*D*—H···*A*
C10—H10*B*···O1^i^	0.99	2.44	3.397 (2)	163

Symmetry code: (i) −*x*, *y*−1/2, −*z*+1/2.
